# Antibacterial Activities of Actinomycete Isolates Collected from Soils of Rajshahi, Bangladesh

**DOI:** 10.4061/2011/857925

**Published:** 2011-09-04

**Authors:** Md. Ajijur Rahman, Mohammad Zahidul Islam, Md. Anwar Ul Islam

**Affiliations:** ^1^Department of Pharmacy, University of Rajshahi, Rajshahi 6205, Bangladesh; ^2^Department of Parasitology, Aichi Medical University, Aichi-ken 480-1195, Japan; ^3^Department of Biology, The Catholic University of America, Washington, DC 20064, USA

## Abstract

This study was performed to isolate actinomycete colonies having antibacterial activity from soil samples collected from different places around Rajshahi, Bangladesh. Thirty actinomycete colonies were isolated in pure culture from five soil samples using Starch-casein-nitrate-agar medium. The isolates were grouped in five color series based on their aerial mycelia color and screened for their antibacterial activity against a range of test bacteria. Sixteen isolates (53.3%) were found to have moderate to high activity against four gram-positive and four gram-negative bacteria. Since many isolates showed inhibitory activity against indicator bacteria, it is suggestive that Bangladeshi soil could be an interesting source to explore for antibacterial secondary metabolites.

## 1. Introduction

Actinomycetes are gram-positive, free-living, saprophytic bacteria, widely distributed in soil, water, and colonizing plants. From the 22,500 biologically active compounds that have obtained form microbes, 45% are produced by actinomycetes, 38% by fungi, and 17% by unicellular bacteria [1]. The species belong to the genus *Streptomyces* constitute 50% of the total population of soil actinomycetes and are well known for producing a variety of bioactive secondary metabolites including antibiotics, immunomodulators, anticancer drugs, antiviral drugs, herbicides, and insecticides [2–4]. *Streptomyces *species are gram-positive, aerobic microorganisms with high DNA G+C contents and produce about half of all known antibiotics from microorganisms. In fact, *Streptomyces *species are the resource of 75% of commercially produced and medically useful antibiotics [5].

Although thousands of antibiotics have been isolated from *Streptomyces*, these represent only a small fraction of the repertoire of bioactive compounds produced [4, 6]. Therefore, isolation of new* Streptomyces* from natural resources and characterization of their secondary metabolites is a valuable endeavor.

Bangladesh, a low-lying riverine country, has a tropical monsoon climate characterized by heavy seasonal rainfall, high temperatures, and high humidity. The alluvial soil of Bangladesh is highly fertile. A few studies have been done so far using Bangladeshi soils to screen for new actinomycetes for new bioactive compounds. *Streptomyces bangladeshensis* was the first report of the discovery of a new species of *Streptomyces,* from soil samples of Bangladesh producing bis-(2-ethylhexyl)-phthalate [7]. Actinomycin D was isolated from a new type strain of *Streptomyces parvulus* strain MARS-17, from soils collected from Rajshahi, Bangladesh [8]. 

This report describes the isolation of actinomycete strains producing antibacterial secondary metabolites from soil samples collected from different places around Rajshahi, Bangladesh.

## 2. Materials and Methods

### 2.1. Soil Samples

Soil samples were collected from the different places of Rajshahi District, Bangladesh (Table 1). Samples were collected from various depth of the earth surface, ranging from layers just beneath the upper surface to 1 meter depth. They were collected in the sterile small plastic tubes and properly labeled with the date of collection. Twenty soil samples were collected within a period of six months (July, 2007–December, 2007). The collected soil samples were dried in a hot air oven at 60–65°C for 3 hours and stored in 4°C until examined.

### 2.2. Isolation of Pure Culture of Actinomycetes

Thirty actinomycete strains were isolated and obtained as pure culture by using standard microbiological method. From each soil sample, 1 gm of dried soil was suspended in 9 mL sterile water, and successive serial dilutions were made by transferring 1mL of aliquots to 2nd test tube containing 9 mL of sterile water, and in this way dilutions up to 10^−4^ were prepared. Each time the contents were vortexed to form uniform suspension. An aliquot of 0.1 mL of each dilution was taken and spread evenly over the surface of starch-casein-nitrate-agar medium supplemented with cycloheximide (100 *μ*g/mL) on 16 cm petridishes. Plates were incubated at 32°C and monitored for 7 days. The colonies were carefully counted by visual observation and c.f.u per gram of soil was determined. Plates those gave 100–150 colonies were chosen for further isolation in pure culture. Suitable colonies those showed *Streptomyces* like appearance under light microscope were recultivated several times for purity. The purified actinomycetes were preserved on yeast-extract-glucose-agar slants at 4°C for two months and at −20°C in the presence of glycerol (15%v/v) for longer periods.

### 2.3. Color Grouping of the Isolates

The color of the aerial mycelia and pigment production by the isolates were determined on yeast-extract-glucose-agar plates after 7 days of incubation at 32°C. The color of the substrate mycelia and those of the soluble pigment were determined according to the National Bureau of Standards Color Chart [9].

### 2.4. Screening of Antimicrobial Activities of Pure Isolates

Preliminary screening for antibiotic activity of the isolates was done by using streak-plating technique on yeast-extract-glucose-agar medium. Each pure isolates were streaked individually on different agar plates in a single line. The plates were then incubated at 32°C for 5 days to allow the isolates to secrete antibiotics into the medium. After the incubation period, the properly diluted test organisms were cross-streaked along the line of fully grown isolates. Each streaking was started near the edge of the plates and streaked toward the *Streptomyces* growth line. The plates were then incubated for 12 hrs at 37°C, and the zone of inhibition was measured using a millimeter scale [10].

### 2.5. Test Organisms

Eight test organisms were used to test the antibiotic activity of the isolates. Four of them were gram-positive and four were gram-negative bacteria. Gram-positive species were *Staphylococcus aureus* ATCC-259233, *Streptococcus agalactiae, Bacillus cereus, *and *Bacillus megaterium* QL-40. Gram-negative strains were *Escherichia coli* FPFC-1407, *Shigella flexneri* AL-30372, *Shigella dysenteriae* AL-35587, and *Shigella sonnei*. They were maintained in nutrient agar slants at 4°C.

## 3. Results and Discussion

**Figure 1:**
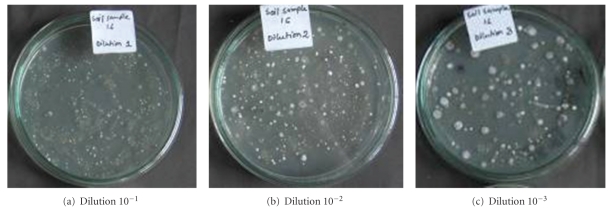
Colonies of actinomycetes appeared on the dilution plates using the soil samples collected from medicinal plant garden.

This study was performed with an aim of isolating actinomycete strains with antimicrobial activities using the selective isolation media. Thirty different actinomycete strains were isolated from 5 soil samples collected from different locations of Rajshahi (Table 1) in the year of 2007. All of these strains were collected by using Starch-casein-nitrate-agar media supplemented with cycloheximide (100 *μ*g/mL) to inhibit fungal growth. This media is very specific for the isolation of actinomycetes, as only organisms (mostly actinomycetes) those are capable of degrading the polymers in the media are able to grow [11]. The colony forming units (c.f.u) were determined by counting the colonies on the dilution plates (Figure 1). Maximum number of colonies (1.45 × 106 c.f.u/gm of soil) were obtained in the soil collected from the cultivated land. This land is used for wheat cultivation. This cultivated land was near to a small lake and was very rich with natural composts which may be the reason for highest count. The result of colony counts is shown in Table 1.

**Figure 2:**
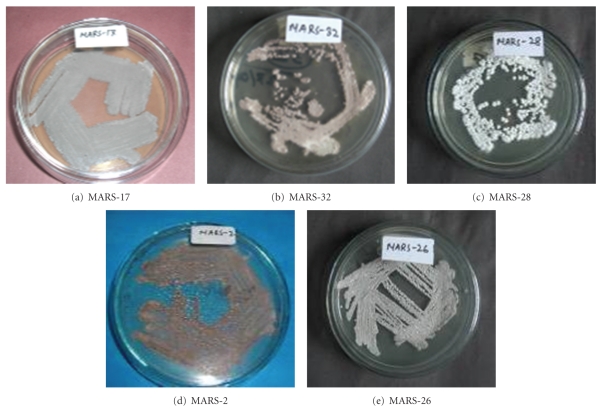
Representative isolates of different color series (a) MARS-17, gray series, (b) MARS-32, brown series, (c) MARS-28, white series, (d)MARS-2, orange series, (e) MARS-26, red series. The isolates were grown on yeast-extract-glucose-agar plates for 7 days at 32°C.

All purified isolates grew on yeast-extract-glucose-agar media showing morphology of typical *Streptomyces*; the colonies were slow growing, aerobic, glabrous or chalky, folded, and with aerial and substrate mycelia of different colors [12]. In addition, all colonies possessed an earthy odor. All of the strains were acid fast negative and gram positive and fitted to the description of genus *Streptomyces* in Bergey's Manual of Systemic Bacteriology. The isolates were categorized into five color series according to their color of the mature sporulated substrate mycelium (Figure 2, Table 2). The gray series isolates were more predominant (33.3% of the total isolates). Out of 30 isolates, two isolates produce soluble pigments in the media (Table 2).

**Figure 3:**
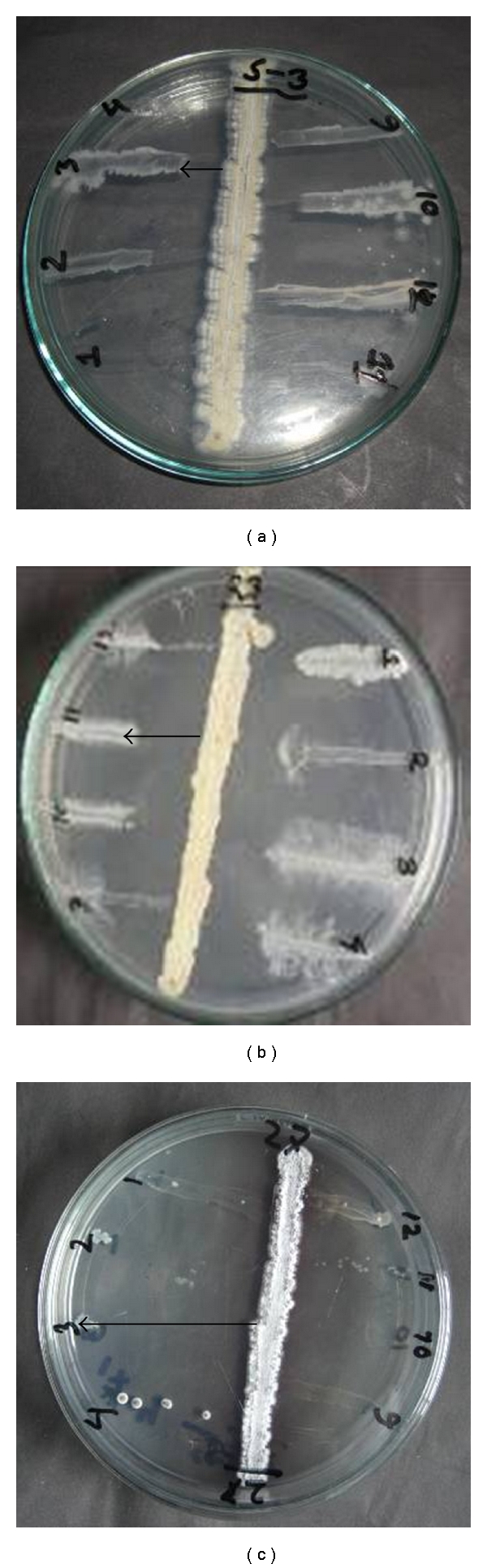
Streak-plating technique to screen the antibacterial activity of isolated* Streptomyces.* The vertical line is the *Streptomyces* strains to be tested and. (*→*) indicates the distance in millimeter (mm) inhibited by the isolates. (a) MARS-3, a low active strain, (b) MARS-23, a moderately active strain, (c) MARS-27, a highly active strain. The perpendicular lines marked with numerical digits are the test organism- *Staphylococcus aureus *ATCC-259233 (1), *Streptococcus agalactiae *(2), *Bacillus cereus *(3), *Bacillus megaterium* QL-40 (4), *Escherichia coli* FPFC-1407 (9), *Shigella flexneri* AL-30372 (10),* Shigella dysenteriae* AL-35587 (11), and *Shigella sonnei* (12).

**Figure 4:**
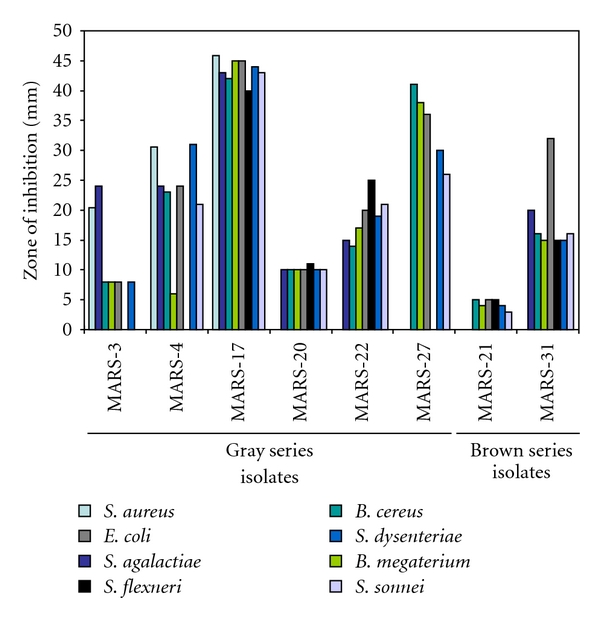
Antibacterial activities of gray and brown series isolates. Only the antibacterial activities of the active isolates have been shown. The zone of inhibition was measured using a millimeter scale from the line of streaking as shown in Figure 3.

**Figure 5:**
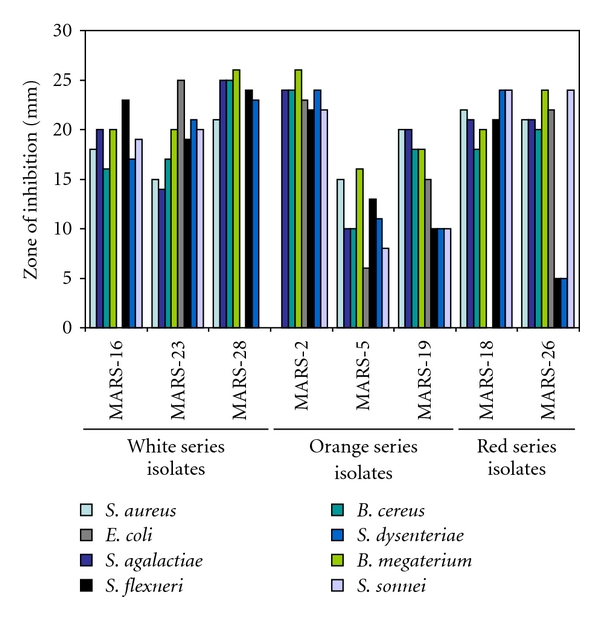
Antibacterial activities of white, orange and red series isolates. Only the antibacterial activities of the active isolates have been shown. The zone of inhibition was measured using a millimeter scale from the line of streaking as shown in Figure 3.

All the isolated actinomycete strains were screened for their antibacterial activity on Yeast-extract-glucose-agar medium using streak-plating technique (Figure 3). A broad spectrum of antibacterial activity was observed in 53.3% (16 out of 30) of the total pure isolates. Percentages of active isolates in between the series were different. About 60.0% of the gray series, 28.0% of brown series, 100.0% of white series, 42.0% orange series, and 66.0% of red series isolates were active against the test bacteria (Figures 4 and 5, Table 3).

The active isolates exhibited different inhibitory patterns against the test organisms. *S. aureus* and *S. sonnei* were inhibited by 13 isolates (81.25%), *S. agalactiae, B. cereus, B. megaterium,* and *S. flexneri* were inhibited by all 16 isolates (100.0%), *E. coli *by 10 isolates (62.5%), and *S. dysenteriae* by 15 isolates (93.75%). MARS-3 could not inhibit *E. coli*, and *S. dysenteriae* (gram-negative bacteria) but exhibited strong activity against* S. aureus*. MARS-4 could not suppress the growth of *E. coli* but strongly inhibited *S. dysenteriae* and *S. sonnei. *MARS-27 were inactive against *S. aureus, S. sonnei,* and *E. coli* but were strongly active against other test bacteria. MARS-21 did not exhibit any activity against *S. aureus*, and *S. *sonnei but showed very weak activity against other test organisms. MARS-16 and MARS-18 were moderate active against all but *E. coli*. MARS-28 could not inhibit *E. coli* and *S. dysenteriae* but was moderately active against the others. MARS-5, MARS-17, MARS-19, MARS-20, MARS-22, MARS-23, MARS-26, and MARS-31 inhibited all test organisms. Among them, the isolate MARS-17 and MARS-27 showed very strong broad-spectrum antibacterial activity (Figures 4 and 5). These two highly active isolates were under gray series. 

 In a study, from the soil samples of Karanjal regions of Sundarbans of Bangladesh, about 55 actinomycetes of different genera were isolated and screened for antibacterial activity [13]. In their screening work, they found that 20 isolates (36.36%) were active against the test organisms. In another study, 356 *Streptomyces *isolates were obtained from soils in the Aegean and East Black Sea regions of Turkey, and 36% of the isolates were found to be active against tested microorganisms [14]. In a recent study performed in 2010 by Dehand et al., [15], the antibacterial activity of *streptomyces* isolates from soil samples of West of Iran was investigated. Out of 150 actinomycetes, only 20 isolates (13.30%) showed activity against the test bacteria.

Comparing the above mentioned results with this study, we can conclude that the soil samples of Rajshahi are rich source of actinomycetes which produce metabolites inhibitory to bacterial pathogens. We found that 53.3% of the isolated colonies were active against the test bacteria. MARS-17 and MARS-27 were very active and showed very large zone of inhibition. Future studies will be done to identify the active isolates up to the species level. The type of antimicrobial agents produced by these isolates will be investigated as well.

## Figures and Tables

**Table 1:** Collection site and depth of soil from where the isolates were collected using Starch-casein-nitrate-agar media.

Date of collection	Collection site	Number of actinomycetes in each gram of soil (c.f.u/gm of dried weight soil)	Isolates
11/02/2007	Cultivated land for wheat production, Rajshahi	1.45 × 10^6^	MARS 1–10
12/02/07	Medicinal Plant Garden, Rajshahi University	1.27 × 10^6^	MARS 11–15
18/03/07	Bank of Pond, Rajshahi University	1.11 × 10^6^	MARS 16–25
03/06/07	Mango orchard, Rajshahi University	1.20 × 10^6^	MARS 26–30

**Table 2:** Color grouping of the isolates.

Color series	Isolate	Color of aerial mycelia	Color of substrate mycelia	Diffusible pigment
Gray series	MARS-1	Light gray	Light gray	ND*
	MARS-3	Light brownish gray	Dark yellowish brown	ND
	MARS-4	Light brownish gray	Grayish red	ND
	MARS-11	Pinkish gray	Dark reddish brown	ND
	MARS-17	Light brownish gray	Grayish yellow	Yellow
	MARS-20	Brownish gray	Darky	ND
	MARS-22	Light gray	Grayish yellow	ND
	MARS-25	Light bluish gray	Tan	ND
	MARS-27	Medium bluish gray	Dusky red	Brown
	MARS-30	Very light gray	Grayish yellow	ND
Brown series	MARS-6	Dark yellowish brown	Grayish yellow	ND
	MARS-8	Dark yellowish brown	Grayish yellow	ND
	MARS-10	Pale reddish brown	Dark yellowish orange	ND
	MARS-12	Pale brown	Grayish orange	ND
	MARS-21	Brownish black	Yellowish gray	ND
	MARS-31	Light brown	Grayish yellow	ND
	MARS-32	Dark reddish brown	Grayish orange	ND
White series	MARS-16	White	Grayish orange	ND
	MARS-23	White	Grayish yellow	ND
	MARS-28	White	Moderate yellow	ND
Orange series	MARS-2	Moderate reddish orange	Grayish orange	ND
	MARS-5	Pale yellowish orange	Grayish orange	ND
	MARS-7	Pale yellowish orange	Dark yellowish orange	ND
	MARS-9	Pale yellowish orange	Grayish orange	ND
	MARS-15	Grayish orange pink	Moderate yellow	ND
	MARS-19	Pale yellowish orange	Moderate yellow	ND
	MARS-29	Pale yellowish orange	Dark yellowish orange	ND
Red series	MARS-18	Pale red	Moderate yellowish brown	ND
	MARS-26	Grayish red	Moderate yellow	ND
	MARS-24	Grayish red	Moderate yellow	ND

*ND: Not detectable.

**Table 3:** Antibacterial activity of the isolates against a wide range of test bacteria.

Bacteria	Zone of inhibition							
	Gram-positive bacteria				Gram-negative bacteria			
Isolates	*S. aureus*	*S. agalactiae*	*B. cereus*	*B. megaterium*	*E. coli*	*S. flexneri*	*S. dysenteriae*	*S. sonnei*
MARS-3	24	08	08	08	0	08	0	10
MARS-4	24	23	06	24	0	31	21	34
MARS-17	43	42	45	45	40	44	43	25
MARS-20	10	10	10	10	11	10	10	10
MARS-22	15	14	17	20	25	19	21	20
MARS-27	0	41	38	36	0	30	26	0
MARS-21	0	5	4	5	5	4	3	0
MARS-31	20	16	15	32	15	15	16	12
MARS-16	18	20	16	20	0	23	17	19
MARS-23	15	14	17	20	25	19	21	20
MARS-28	21	25	25	26	0	24	23	0
MARS-2	0	24	24	26	23	22	24	22
MARS-5	15	10	10	16	06	13	11	08
MARS-19	20	20	18	18	15	10	10	10
MARS-18	22	21	18	20	0	21	24	24
MARS-26	21	21	20	24	22	05	05	24
